# Supplementary material for: Comparison between two- and three-dimensional scoring of zebrafish response to psychoactive drugs: identifying when three-dimensional analysis is needed
Source: PeerJ. 2019 Oct 16;7:e7893. doi: 10.7717/peerj.7893 (PMC6800527; doi:10.7717/peerj.7893)
Supplement: Supplemental Information 1 [file peerj-07-7893-s001.docx]

**Supplementary material**

*Effect of condition, time, and their interaction, irrespective of view*

**Citalopram**

Average speed: Average speed did not vary among conditions (condition: *F*_3,55_ = 0.54, *P* = 0.655). While it did not seem to change over time (time bins: *F*_5,275_ = 0.91, *P* = 0.449), it selectively increased over the first few minutes in 30 mg/L citalopram subjects (time bins × condition: *F*_15,275_ = 2.21, *P* = 0.006; *t*_289.0_ *=* 2.77, *P* < 0.027), but declined in 100 mg/L (*t*_289.0_ *=* 3.52, *P* = 0.002).

Average peak speed: Average peak speed did not vary among conditions (condition: *F*_3,55_ = 0.42, *P* = 0.74). However, it decreased significantly over time (time bins: *F*_5,275_ = 3.46, *P* = 0.005; *t*_278.8_ > 2.71, *P* < 0.031), where we registered a decrease in 50 mg/L and 100 mg/L citalopram conditions (time × condition: *F*_15,275_ = 1.77, *P* = 0.038; *t*_278.8_ *>* 2.97, *P* < 0.015).

Average angular speed: Average angular speed did not vary among conditions (condition: *F*_3,55_ = 0.09, *P* = 0.966). It also did not significantly vary with time (time bins: *F*_5,275_ = 2.02, *P* = 0.076). However, it declined in time in control as well as in 30 mg/L and 50 mg/L citalopram conditions, but not in 100 mg/L condition (time bins × condition: *F*_15,275_ = 2.20, *P* = 0.007; *t*_491.4_ > 2.64, *P* < 0.037).

Average peak angular speed: Average peak angular speed did not vary among conditions (condition: *F*_3,55_ = 0.40, *P* = 0.756). It also failed to significantly vary with time (time bins: *F*_5,275_ = 2.10, *P* = 0.065). However, it significantly decreased over time in the 30 mg/L citalopram condition (time bins × condition: *F*_15,275_ = 2.01, *P* = 0.015; *t*_459.6_ > 2.64, *P* < 0.037).

Average acceleration: Average acceleration did not vary among conditions (condition: *F*_3,55_ = 0.77, *P* = 0.515) nor with time and condition (time bins × condition: *F*_15,275_ = 0.81, *P* = 0.665). It also failed to reach significant variation with time (time bins: *F*_5,275_ = 1.97, *P* = 0.084.

Average peak acceleration: Average peak acceleration did not vary among conditions (condition: *F*_3,55_ = 0.82, *P* = 0.486). However, it significantly decreased over time (time bins: *F*_5,275_ = 4.36, *P* = 0.001; *t*_297.2_ *>* 3.45, *P* < 0.030), but this did not depend on condition (time bins × condition: *F*_15,275_ = 0.96, *P* = 0.500).

Wall following: Wall following did not vary among conditions (condition: *F*_3,55_ = 0.99, *P* = 0.403), nor did it vary over time (time bins: *F*_5,275_ = 1.91, *P* = 0.092), nor with time and condition (time × condition: *F*_15,275_ = 1.19, *P* = 0.278).

Position in the water column (proportion of time spent in the top half): The time spent in the top half of the water column did not vary among conditions (condition: *F*_3,55_ = 1.16, *P* = 0.333) (figure 5h), nor did it change over time (time bins: *F*_5,275_ = 1.32, *P* = 0.254) or with time and condition (time bins × condition: *F*_15,275_ = 1.00, *P* = 0.451).

Freezing: The time spent freezing did not vary among conditions (condition: *F*_3,55_ = 0.39, *P* = 0.756) and it did not change over time (time bins: *F*_5,275_ = 0.04, *P* = 0.433). However, it significantly decreased over time in the 30 mg/L citalopram condition (time bins × condition: *F*_15,175_ = 1.71, *P* = 0.049; *t*_278.1_ > 2.74, *P* < 0.029).

**Ethanol**

Average speed: Average speed did not vary among conditions (condition: *F*_3,52_ = 1.91, *P* = 0.139), nor did it vary over time (time bins: *F*_5,260_ = 0.38, *P* = 0.863) or with time and condition (time bins × condition: *F*_15,260_ = 1.39, *P* = 0.151).

Average peak speed: Average peak speed did not vary among conditions (condition: *F*_3,52_ = 1.50, *P* = 0.225). However, it significantly decreased over time (time bins: *F*_5,260_ = 2.68, *P* = 0.022; *t*_262.6_ *=* 2.99, *P* = 0.014), but was not influenced by the treatment (time bins × condition: *F*_15,260_ = 1.10, *P* = 0.34).

Average angular speed: Average angular speed did not vary among conditions (condition: *F*_3,52_ = 0.88, *P* = 0.456), nor did it vary with time (time bins: *F*_5,260_ = 1.15, *P* = 0.139). However it decreased over time in the 0.5% and 1.0% ethanol conditions (time bins × condition: *F*_15,260_ = 1.73, *P* = 0.046; *t*_471.1_ *>* 2.69, *P* < 0.033).

Average peak angular speed: Average peak angular speed did not vary among conditions (condition: *F*_3,52_ = 0.563, *P* = 0.600), nor did it vary with time (time bins: *F*_5,260_ = 2.03, *P* = 0.075) or as a function of time and condition (time bins × condition: *F*_15,260_ = 2.33, *P* = 0.091).

Average acceleration: Average acceleration varied among conditions (condition: *F*_3,52_ = 2.86, *P* = 0.046), but post-hoc tests revealed no difference with respect to control subjects. The variation with time failed to reach significance (time bins: *F*_5,260_ = 2.23, *P* = 0.052), and it did not vary with time and condition (time bins × condition: *F*_15,260_ = 1.25, *P* = 0.233).

Average peak acceleration: Average peak acceleration varied among conditions (condition: *F*_3,52_ = 3.02, *P* = 0.038), but post-hoc tests revealed no difference with respect to the control condition. We also registered a significant decrease with time (time bins: *F*_5,260_ = 5.60, *P* < 0.001; *t*_277.3_ *=* 3.56, *P* < 0.021), but this was not influenced by condition (time bins × condition: *F*_15,260_ = 1.44, *P* = 0.128).

Wall following: Wall following failed to reach a statistically significant variation among conditions (condition: *F*_3,52_ = 2.63, *P* = 0.060). It was found to vary with time (time bins: *F*_5,260_ = 2.40, *P* = 0.038), but post-hoc tests failed to reveal any significant difference. This was not influenced by the condition (time bins × condition: *F*_15,260_ = 0.88, *P* = 0.587).

Position in the water column (proportion of time spent in the top half: The time spent in the top half of the water column did not vary among conditions (condition: *F*_3,52_ = 2.31, *P* = 0.096) (figure 6h). However, it significantly increased with time (time bins: *F*_5,260_ = 6.01, *P* < 0.001; *t*_260_ > 3.07, *P* < 0.011), where it significantly increased over time in the ethanol 1.0% condition (time bins × condition: *F*_15,260_ = 1.98, *P* = 0.017; *t*_260_ > 3.32, *P* < 0.005).

Freezing: Fish in 0.5% and 1.0% ethanol conditions were found to freeze significantly more than control subjects (condition: *F*_3,52_ = 3.39, *P* = 0.025; *t*_86.1_ *>* 2.49; *P* < 0.040). However, the time spent freezing did not vary over time (time bins: *F*_5,260_ = 0.69, *P* = 0.630), nor with time and condition (time bins × condition: *F*_15,260_ = 1.35, *P* = 0.171).

*Effect of condition, time, and their interaction, based on solely 3D data*


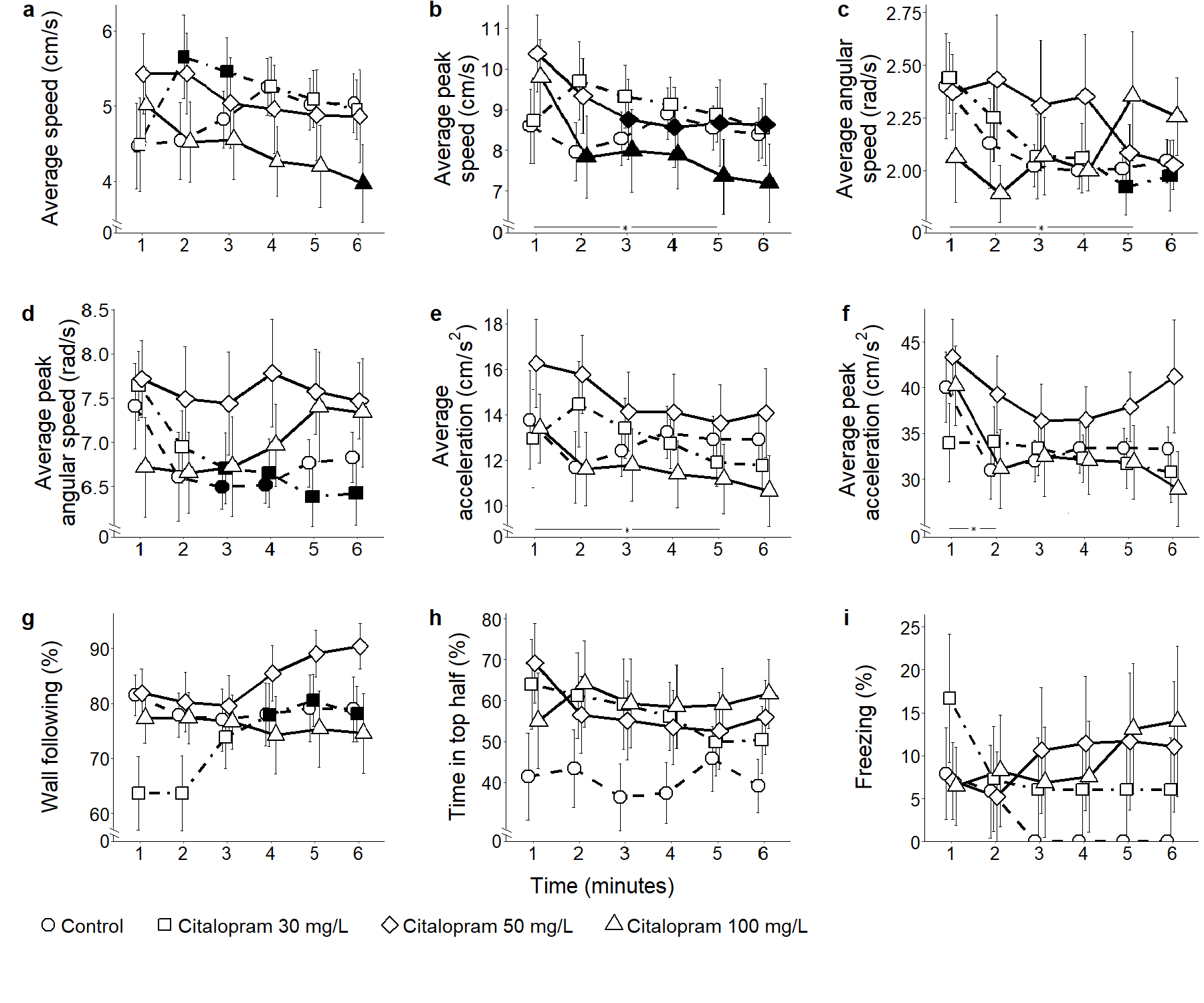


**Figure S1.** Mean $\pm$ standard error for a) average speed, b) average peak speed, c) average angular speed, d) average peak angular speed, e) average acceleration, f) average peak acceleration, g) proportion of time spent within 3 cm of walls, h) proportion of time spent in the top half of the tank, and i) proportion of time spent freezing, over 6-minute trials for control and each citalopram condition, computed from 3D reconstructed trajectories. Data were analyzed through a repeated measures ANOVA for split-plot designs. Filled symbols denote a significant difference (*P* < 0.05) from the first minute within each condition. Horizontal bar denotes a significant overall difference over time. Filled symbols in the top right corner of each panel denote a significant overall difference with respect to the control condition.

**Citalopram (figure S1)**

Average speed: Average speed did not vary among conditions (condition: *F*_3,55_ = 0.50, *P* = 0.683) (figure S1a). It also did not significantly change over time (time bins: *F*_5,275_ = 0.94, *P* = 0.457), although we registered an increase over the first few minutes for 30 mg/L (time bins × condition: *F*_15,275_ = 2.18, *P* = 0.007; *t*_275_ > 2.79, *P* < 0.025), and a decrease over time for 100 mg/L citalopram subjects (*t*_275_ > 2.90, *P* < 0.019).

Average peak speed: Average peak speed did not vary among conditions (condition: *F*_3,55_ = 0.41, *P* = 0.749) (figure S1b). However, it decreased over time (time bins: *F*_5,275_ = 3.36, *P* = 0.006; *t*_275_ > 3.22; *P* < 0.065), where we registered a decline for 50 mg/L and 100 mg/L citalopram conditions (time bins × condition: *F*_15,275_ = 1.73, *P* = 0.044; *t*_275_ *>* 2.65; *P* < 0.037).

Average angular speed: Average angular speed did not vary among conditions (condition: *F*_3,55_ = 0.24, *P* = 0.868) (figure S1c). However, it significantly decreased over time (time bins: *F*_5,275_ = 2.36, *P* = 0.041), where we registered a decline for 30 mg/L citalopram administration (time bins × condition: *F*_15,275_ = 1.87, *P* = 0.026; *t*_275_ *>* 2.73, *P* < 0.030).

Average peak angular speed: Average peak angular speed did not vary among conditions (condition: *F*_3,55_ = 0.91, *P* = 0.443) (figure S1d). It also failed to reach a statistically significant variation with time (time bins: *F*_5,275_ = 2.19, *P* = 0.056), although we registered a decline for control and 30 mg/L citalopram conditions (time bins × condition: *F*_15,275_ = 1.71, *P* = 0.050; *t*_275_ *=* 2.54, *P* < 0.049).

Average acceleration: Average acceleration did not vary among conditions (condition: *F*_3,55_ = 0.76, *P* = 0.523) (figure S1e). It significantly decreased with time (time bins: *F*_5,275_ = 2.29, *P* = 0.046; *t*_275_ *>* 2.73, *P* < 0.030), but this was not influenced by the condition (time bins × condition: *F*_15,275_ = 0.83, *P* = 0.644).

Average peak acceleration: Average peak acceleration did not vary among conditions (condition: *F*_3,55_ = 0.86, *P* = 0.466) (figure S1f). It significantly decreased with time (time bins: *F*_5,275_ = 4.82, *P* < 0.001; *t*_275_ *=* 3.61, *P* < 0.018), but this was not influenced by the condition (time bins × condition: *F*_15,275_ = 0.92, *P* = 0.544).

Wall following: Wall following did not vary among conditions (condition: *F*_3,55_ = 1.25, *P* = 0.301) (figure S1g), but it did change over time (time: *F*_5,275_ = 2.41, *P* = 0.037). In particular, it increased in the 30 mg/L citalopram condition (time bins × condition: *F*_5,275_ = 1.71, *P* = 0.049; *t*_275_ *>* 3.04, *P* < 0.012).

Position in the water column (proportion of time spent in the top half): The time spent in the top half of the water column did not vary among conditions (condition: *F*_3,55_ = 1.16, *P* = 0.333) (figure S1h), nor did it change over time (time bins: *F*_5,275_ = 1.32, *P* = 0.254) or with time and condition (time bins × condition: *F*_15,275_ = 1.00, *P* = 0.451).

Freezing: The time spent freezing did not vary among conditions (condition: *F*_3,55_ = 0.39, *P* = 0.754) (figure S1i), nor did it change over time (time bins: *F*_5,275_ = 0.83, *P* = 0.530). It also failed to reach a statistically significant variation with respect to time and condition (time bins × condition: *F*_15,275_ = 1.62, *P* = 0.069).


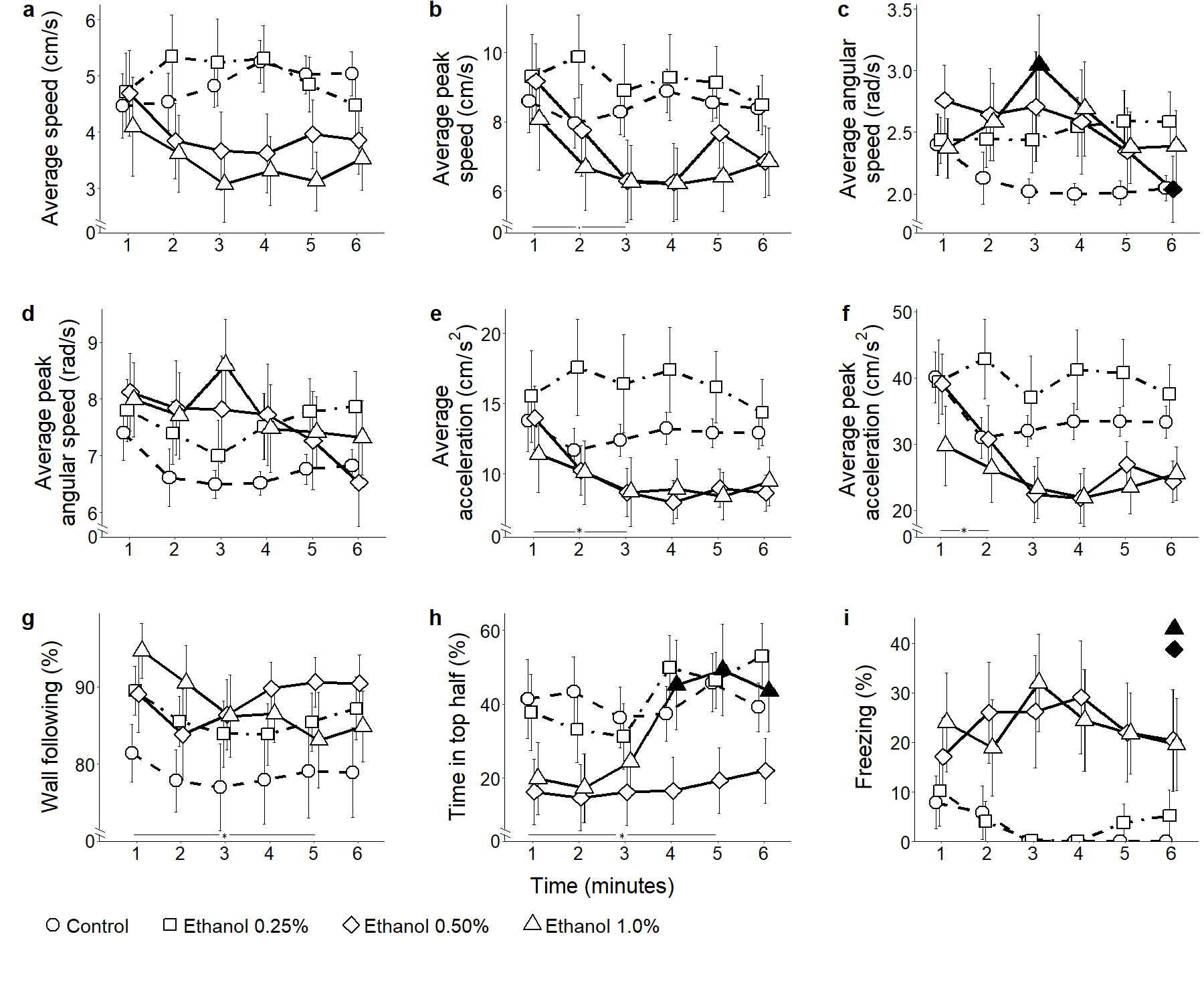


**Figure S2.** Mean $\pm$ standard error for a) average speed, b) average peak speed, c) average angular speed, d) average peak angular speed, e) average acceleration, f) average peak acceleration, g) proportion of time spent within 3 cm of walls, h) proportion of time spent in the top half of the tank, and i) proportion of time spent freezing, over 6-minute trials for control and each ethanol condition, computed from 3D reconstructed trajectories. Data were analyzed through a repeated measures ANOVA for split-plot designs. Filled symbols denote a significant difference (*P* < 0.05) from the first minute within each condition. Horizontal bar denotes a significant overall difference over time. Filled symbols in the top right corner of each panel denote a significant overall difference with respect to the control condition.

**Ethanol (figure S2)**

Average speed: Average speed did not vary among conditions (condition: *F*_3,52_ = 1.93, *P* = 0.137) (figure S2a), nor did it vary over time (time: *F*_5,260_ = 0.45, *P* = 0.816) or with time and condition (time bins × condition: *F*_15,260_ = 1.46, *P* = 0.121).

Average peak speed: Average peak speed did not vary among conditions (condition: *F*_3,52_ = 1.51, *P* = 0.22) (figure S2b). We registered a decrease over time (time bins: *F*_5,260_ = 2.84, *P* = 0.016; *t*_260_ *>* 2.72, *P* < 0.030), although we did not identify a variation with time and condition (time bins × condition: *F*_2,260_ = 1.16, *P* = 0.306).

Average angular speed: Average angular speed did not vary among conditions (condition: *F*_3,52_ = 0.95, *P* = 0.426) (figure S2c), nor did it change with time (time bins: *F*_5,260_ = 1.93, *P* = 0.091).

Average peak angular speed: Average peak angular speed did not vary among conditions (condition: *F*_3,52_ = 0.68, *P* = 0.570) (figure S2d), nor did it vary with time (time bins: *F*_5,260_ = 1.95, *P* = 0.087) or as a function of both time and condition (time bins × condition: *F*_15,260_ = 1.54, *P* = 0.091).

Average acceleration: Average acceleration did not vary among conditions (condition: *F*_3,52_ = 2.77, *P* = 0.051) (figure S2e). It significantly decreased with time (time bins: *F*_5,260_ = 2.42, *P* = 0.036; *t*_260_ *>* 2.67, *P* < 0.032), although such a decline was not influenced by the condition (time bins × condition: *F*_15,260_ = 2.42, *P* = 0.173).

Average peak acceleration: Although condition was found to be significant (condition: *F*_3,52_ = 3.02, *P* = 0.038), post-hoc tests failed finding differences between treatments and control (figure S2f). However, it decreased with time (time bins: *F*_5,260_ = 6.28, *P* < 0.001; *t*_260_ > 2.58, *P* < 0.045), but this did not depend on the treatment (time bins × condition: *F*_15,260_ = 1.59, *P* = 0.075).

Wall following: Wall following did not vary among conditions (condition: *F*_3,52_ = 1.38, *P* = 0.258) (figure S2g). It did not change with time (time bins: *F*_5,260_ = 1.56, *P* = 0.172), nor did it vary depending on time and condition (time bins × condition: *F*_15,260_ = 0.973, *P* = 0.759).

Position in the water column (proportion of time spent in the top half): The time spent in the top half of the water column did not vary among conditions (condition: *F*_3,52_ = 2.23, *P* = 0.096) (figure S2h). However, we observed that the time spent in the top half increased over time (time bins: *F*_5,260_ = 6.01, *P* < 0.001), selectively in the 1.0% ethanol condition (time bins × condition: *F*_15,260_ = 1.98, *P* = 0.017; *t*_260_ *=* 3.32, *P* = 0.005). Finally, it was higher in males (time bins: *F*_1,52_ = 11.01, *P* = 0.002).

Freezing: Fish treated at 0.5% and 1.0% ethanol concentrations conditions were found to freeze more than control subjects (condition: *F*_3,52_ = 3.33, *P* = 0.027; *t*_86.3_ *>* 2.49; *P* < 0.040) (figure S2i). However, we did not observe variations over time (time bins: *F*_5,260_ = 0.56, *P* = 0.73) or with time and condition (time bins × condition: *F*_15,260_ = 1.35, *P* = 0.175).
